# Antipsychotics function as epigenetic age regulators in human neuroblastoma cells

**DOI:** 10.1038/s41537-022-00277-1

**Published:** 2022-08-29

**Authors:** Jianbin Du, Yutaka Nakachi, Ayaka Fujii, Shinya Fujii, Miki Bundo, Kazuya Iwamoto

**Affiliations:** Department of Molecular Brain Science, Graduate School of Medical Sciences, Kumamoto University, Kumamoto, Japan

**Keywords:** Schizophrenia, Genetics of the nervous system

## Abstract

Recent epigenetic age studies suggested accelerated aging in schizophrenia. Although antipsychotics may modulate epigenetic age, direct estimation of their roles was impeded when tissues derived from patients were used for analysis. By using a cellular model, we found that antipsychotics generally worked as epigenetic age regulators in vitro.

Schizophrenia is proposed to be a syndrome of accelerated aging in the body, which is partly the cause of the shortened lifespan of patients with schizophrenia^1–3^. Morphological, functional and transcriptomic changes similar to those in aged brains have been found in the brains of patients with schizophrenia^4–7^.

The biological age of a tissue can be accurately determined using the DNA methylation levels of aging-associated CpGs^8–10^. For example, Horvath’s clock, the most widely accepted multitissue epigenetic age estimator, uses DNA methylation levels at 353 CpG sites^8^. Various DNA methylation-based age estimators have been developed to maximize the specificity and sensitivity in different conditions^11^. Most of them are developed based on the blood samples but can still achieve reasonable accuracy in other tissues including the brain. Significant differences between epigenetic age and chronological age have been reported in neurodegenerative diseases such as Parkinson’s^12^ and Huntington’s^13^ diseases and bipolar disorder^14,15^. In schizophrenia, recent large-scale studies suggested accelerated aging^16,17^, although others reported no alterations^18–20^ or delayed epigenetic age acceleration^21^. The inconsistency was possibly due to the complexity of the disease course, medication status, and differences in the cohorts as well as differences in the age estimators used.

We previously performed comprehensive DNA methylation assays of human neuroblastoma cells cultured with various antipsychotics and revealed that the antipsychotics induced similar epigenetic signatures^22–24^. Although experiments using cell lines had several limitations in that they acquired immortal, cancer-like characteristics and did not reflect complex synaptic networks in the brain, they provided the ideal opportunity to test the effects of antipsychotics in vitro. Here, we tested whether antipsychotics affected the epigenetic age of cells.

We calculated the epigenetic ages of human neuroblastoma SK-N-SH cells cultured with antipsychotics, including haloperidol (HAL), risperidone (RIS), blonanserin (BL), and perospirone (PE), for 8 days. For each antipsychotic, two doses (high and low), which were determined based on their effective blood concentrations, were tested with each DNA methylation assay. The assays using HAL and RIS^22^ and those using BL and PE^23,24^ were conducted over different periods of time. Although we used the same source of cell lines and employed the same experimental protocol, principal component analysis suggested batch effect differences between datasets (Fig. S1). Therefore, we performed the statistical analyses separately.

We compared the average epigenetic age between the cells cultured with antipsychotics and the cells cultured as controls with six epigenetic age estimators. We regarded controls as the cells cultured with the solvent of the antipsychotics, dimethyl sulfoxide (DMSO), and first combined the data of the two doses for each antipsychotic for comparison. We found that all estimators except for MiAge^25^ detected a significant decrease in the epigenetic age of cells in at least one antipsychotic culture condition compared to the control culture condition (Figs. 1, 2, and Tables S1, 2). Among the estimators, the SkinBlood estimator^26^ detected a significant decrease in the epigenetic age of cells in all antipsychotic groups, with HAL showing the strongest decelerating effect. We then compared the differences between the dose subgroups and the controls (Figs. 1, 2, and Tables S3–6). As expected, the SkinBlood estimator detected consistent decelerations except in the low-dose BL condition. Remarkably, a high dose of HAL showed the most consistent and the largest decelerations across different estimators, including MiAge, and showed an increase in analyses with DNAmTL, which estimates telomere length^27^. Apart from the SkinBlood estimator, other estimators detected deceleration effects differently. The Hannum^10^ and Weidner clocks^28^ detected effects of HAL and RIS, whereas the Vidal-Bralo clock^29^ detected effects of BL and PE, and the Horvath clock detected an effect of PE. Although the different estimators showed some correlations in our data (Fig. S2), these results suggested that using multiple estimators and selecting the appropriate estimators are critical. We observed that the effect of antipsychotics on epigenetic age differed depending on the concentration. Because we tested only two doses for one culture period for each drug, we may have missed the optimal condition for DNA methylation age estimation. Previous studies suggested that SkinBlood closely tracked replicative senescence and is more suitable for cultures of various cell types, such as fibroblasts and lymphoblastoid cells, than multitissue estimators^26^. Although further validation is needed, SkinBlood may be a suitable estimator for neuroblastoma cells.

**Fig. 1:**
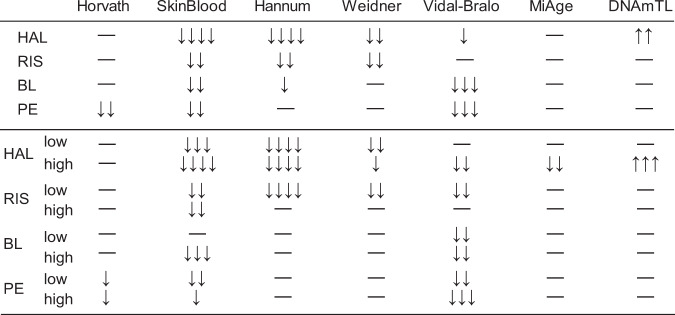
Summary of the effects of antipsychotics on the epigenetic age of neuroblastoma cells. Upper rows indicate the results of combined doses. HAL haloperidol group, RIS risperidone group, BL blonanserin group, PE perospirone group. low: low-dose drug group, high: high-dose drug group. Arrows indicate the direction of change. −, ↓, ↓↓, ↓↓↓, and ↓↓↓↓ indicate *p* ≥ 0.1; *p* < 0.1; *p* < 0.05; *p* < 0.005; *p* < 0.0005, respectively. Means, differences and other statistics are listed in Supplementary Tables S1 to S6.

**Fig. 2:**
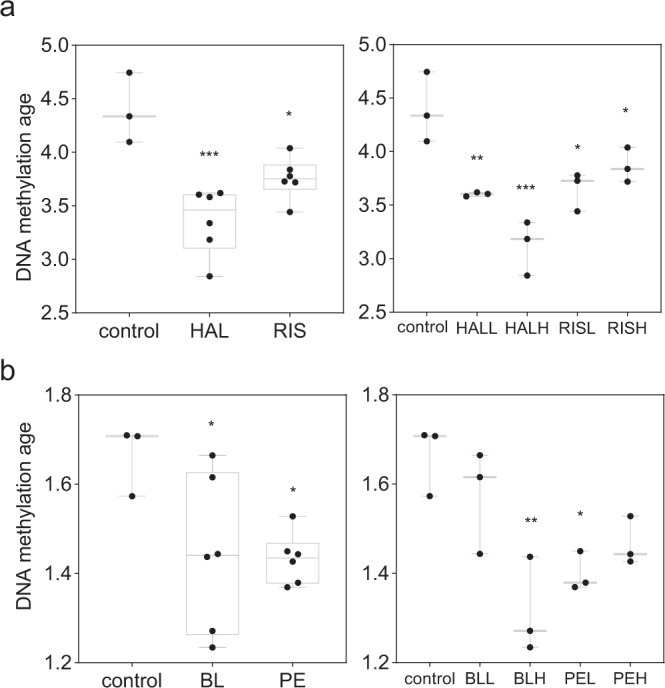
Epigenetic age measured by the SkinBlood estimator. **a** Comparison between the control and either the HAL group or the RIS group. **b** Comparison between the control and either the PE group or the BL group. Left: combined analysis of both low (L)- and high (H) doses. The numbers of samples for the control and drug groups were 3 and 6, respectively. Right: separate analysis of the same dataset by dose. The number of samples in each group was 3. *, **, and *** indicate *p* < 0.05, *p* < 0.005, and *p* < 0.0005, respectively.

We showed that antipsychotics generally demonstrated epigenetic age deceleration effects in the cell culture model. Similar effects have been reported in patients with bipolar disorder treated with mood stabilizers^30^ and those with schizophrenia treated with clozapine^16^. Interestingly, a large-scale analysis of the GTEx^31^ and CMap^32^ transcriptome data identified HAL as an antiaging compound that induced a youthful transcriptional state^33^. Although the molecular mechanism of the effect of HAL on aging remains unclear, considering that our and previous findings were obtained from cell lines and natural human tissues that were not related to dopaminergic neurons, the effect of HAL on aging is likely to be independent of dopamine D2 receptor antagonism. We examined the expression levels of candidate receptors for HAL including dopamine (*DRD1, DRD2, DRD3, DRD4*, and *DRD5*), serotonin (*HTR1A*, *HTR2A*, *HTR2C, HTR6*, and *HTR7*), adrenergic (*ADRA1A, ADRA2A, ADRA2B*, and *ADRA2C*), histamine (*HRH1*), muscarinic (*CHRM1*) and sigma (*SIGMAR1* and *SIGMAR2*) receptors using the DepMap database (10.6084/m9.figshare.15160110.v2). Although the SK-N-SH cell line does not or very weakly express most of these receptors, including *DRD2*, it expresses the sigma-1 receptor (*SIGMAR1*) at a modest level. The sigma-1 receptor is a transmembrane protein located at the endoplasmic reticulum and modulates calcium signaling via inositol 1,4,5-trisphosphate receptors^34^. HAL is an antagonist of the sigma-1 receptor and is also suggested to irreversibly inactivate sigma-1 receptor through a HAL metabolite^35,36^. In the mouse neuroblastoma cell line, NG-108, HAL was suggested to play a role in neuronal differentiation via sigma-1 blockage^37^. Therefore, we speculated that sigma-1 antagonism might be one of the possible mechanisms. Further studies will provide another direction for exploring the aging-related pharmacological effects of antipsychotics.

## Methods

We utilized two previously published DNA methylation datasets, which used an Illumina HumanMethylation450 BeadChip (Illumina, Inc. San Diego, CA, USA). In brief, human neuroblastoma SK-N-SH cells were cultured with antipsychotics for 8 days. After culture, genome-wide DNA methylation analysis was conducted. Dataset 1 (GSE185973) contains HAL and RIS as well as controls. Dataset 2 (GSE192626) contains BL and PE as well as controls. We used two different doses of each antipsychotic (low and high concentrations, respectively: 1 µM and 10 µM for HAL, 3 µM and 30 µM for RIS, 1.4 nM and 13.6 nM for BL, and 10.5 nM and 105.5 nM for PE). Antipsychotics were dissolved in DMSO. Each experimental group included three independent samples.

We utilized 7 DNA methylation estimators applying Horvath’s online calculator (http://dnamage.genetics.ucla.edu/) automatically or R (https://www.bioconductor.org/) manually. Six of these estimators were epigenetic age estimators^8,10,26,28,29^. The other estimator was a telomere length estimator (DNAmTL)^27^. To test for differences in epigenetic age, one-way analysis of variance followed by Dunnett’s test was employed. Differences were considered significant at *P* < 0.05. All statistical analyses were performed using GraphPad Prism (version 8, GraphPad Prism Software Inc. San Diego, CA, USA).

## Supplementary information

Supplementary Figures

Supplementary Tables

## Data Availability

The DNA methylation data used in this study were available through GSE192626 and GSE185973.
