# Supplementary material for: Antipsychotics function as epigenetic age regulators in human neuroblastoma cells
Source: Schizophrenia (Heidelb). 2022 Aug 29;8(1):69. doi: 10.1038/s41537-022-00277-1 (PMC9424249; doi:10.1038/s41537-022-00277-1)
Supplement: Supplementary file 1 — Supplementary Figures [file 41537_2022_277_MOESM1_ESM.pdf]

## Supplementary Figures

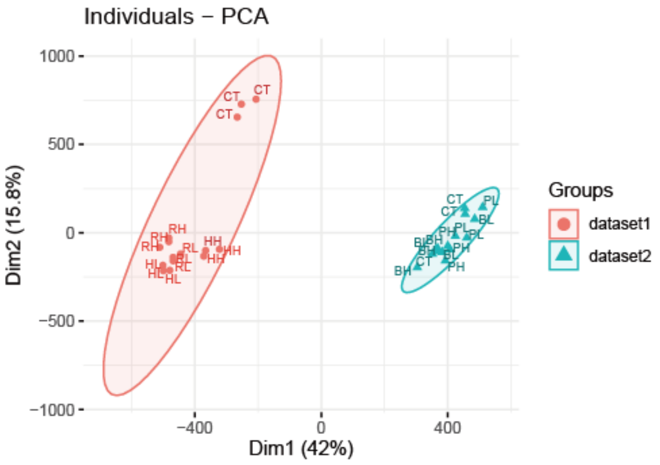


**Supplementary Figure 1**. Principal component analysis of dataset 1 and dataset 2. Samples' ID is marked red or blue color by the batch they belong to. CT, control; RH, risperidone (high-dose); RL, risperidone (low-dose), HH, haloperidol (high-dose); HL, haloperidol (low-dose); PH, perospirone (high-dose); PL, perospirone (low-dose); BH, blonanserin (high-dose); BL, blonanserin (low-dose).

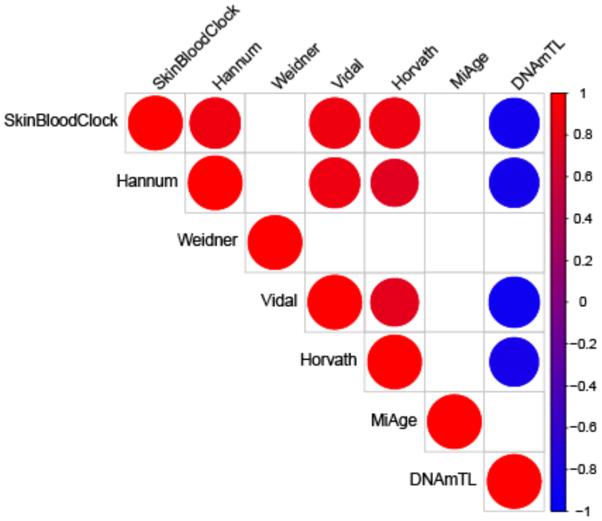


**Supplementary Figure 2**. Spearman correlation matrix between epigenetic age for all pairwise combinations. The color of each circle indicates the direction and strength of correlation coefficient. The size represents the significance ($P<0.05$) of the correlation. The blank cells indicate no significance.
