# Supplementary material for: Antipsychotics function as epigenetic age regulators in human neuroblastoma cells
Source: Schizophrenia (Heidelb). 2022 Aug 29;8(1):69. doi: 10.1038/s41537-022-00277-1 (PMC9424249; doi:10.1038/s41537-022-00277-1)
Supplement: Supplementary file 2 — Supplementary Tables [file 41537_2022_277_MOESM2_ESM.pdf]

Supplementary Tables

Table S1. Effect of antipsychotics on epigenetic age of neuroblastoma cells (combined analysis of dataset 1).

	mean epigenetic age			HAL vs. control		RIS vs.control	
	control	HAL	RIS	difference (95% CI)	adjusted P value	difference (95% CI)	adjusted P value
Horvath	34.89	35.02	36.69	0.14 (-2.49 to 2.76)	0.9858	1.80 (-0.83 to 4.42)	0.1865
SkinBlood	4.39	3.36	3.76	-1.03 (-1.50 to -0.56)	**0.0003**	-0.63 (-1.11 to -0.16)	**0.0107**
Hannum	33.24	29.72	31.6	-3.52 (-4.97 to -2.08)	**0.0001**	-1.63 (-3.08 to -0.19)	**0.0278**
Weidner	19.07	15.59	16.09	-3.49 (-5.82 to -1.16)	**0.0055**	-2.98 (-5.31 to -0.65)	**0.0145**
Vidal-Bralo	62.25	60.82	61.23	-1.42 (-3.09 to 0.24)	0.0929	-1.02 (-2.68 to 0.65)	0.2469
MiAge	663.2	644.8	665	-18.45 (-41.80 to 4.90)	0.1221	1.74 (-21.61 to 25.09)	0.9718
DNAmTL	6.47	6.61	6.51	0.13 (0.02 to 0.25)	**0.0206**	0.03 (-0.08 to 0.15)	0.6544

Table S2. Effect of antipsychotics on epigenetic age of neuroblastoma cells (combined analysis of dataset 2).

	mean epigenetic age			BL vs. control		PE vs. control	
	control	BL	PE	difference (95% CI)	adjusted P value	difference (95% CI)	adjusted P value
Horvath	26.61	26.1	23.9	-0.51(-2.69 to 1.66)	0.7666	-2.71 (-4.89 to -0.54)	**0.0167**
SkinBlood	1.66	1.45	1.43	-0.22 (-0.43 to -0.01)	**0.045**	-0.23 (-0.45 to -0.02)	**0.0352**
Hannum	25.12	23.13	23.43	-1.99 (-4.00 to 0.03)	0.0531	-1.68 (-3.70 to 0.33)	0.1012
Weidner	16.83	16.16	16.4	-0.67 (-2.74 to 1.40)	0.6198	-0.43 (-2.51 to 1.64)	0.8066
Vidal-Bralo	56.58	55.33	55.25	-1.25 (-1.96 to -0.53)	**0.0018**	-1.33 (-2.04 to -0.61)	**0.0011**
MiAge	669.8	661.1	672.6	-8.67 (-33.69 to 16.35)	0.5848	2.81 (-22.20 to 27.83)	0.9376
DNAmTL	7.22	7.24	7.24	0.02 (-0.05 to 0.09)	0.6473	0.02 (-0.04 to 0.09)	0.5506

Table S3. Effect of haloperidol dose on epigenetic age of neuroblastoma cells.

	mean epigenetic age			difference (95% CI), adjusted P value	
	control	HAL-low	HAL-high	HAL-low vs. control	HAL-high vs. control
Horvath	34.89	36.27	33.78	1.38 (-1.76 to 4.52), 0.5444	-1.11 (-4.25 to 2.03), 0.7055
SkinBlood	4.39	3.6	3.12	-0.79 (-1.30 to -0.28), **0.0039**	-1.27 (-1.78 to -0.76), **0.0001**
Hannum	33.24	30.24	29.2	-3.00(-4.00 to -2.00), **<0.0001**	-4.04 (-5.04 to -3.04), **<0.0001**
Weidner	19.07	14.97	16.2	-4.10 (-7.13 to -1.07), **0.0095**	-2.87 (-5.90 to 0.16), 0.0637
Vidal-Bralo	62.25	61.36	60.28	-0.88 (-2.60 to 0.83), 0.4226	-1.96 (-3.68 to -0.25), **0.0254**
MiAge	663.2	657.1	632.4	-6.14 (-31.72 to 19.45), 0.8916	-30.77(-56.35 to -5.18), **0.0192**
DNAmTL	6.47	6.55	6.66	0.08 (-0.02 to 0.19), 0.1241	0.19 (0.08 to 0.29), **0.0013**

Table S4. Effect of risperidone dose on epigenetic age of neuroblastoma cells.

	mean epigenetic age			difference (95% CI), adjusted P value	
	control	RIS-low	RIS-high	RIS-low vs. control	RIS-high vs. control
Horvath	34.89	36.51	36.85	1.63 (-151 to 4.77), 0.4153	1.97 (-1.17 to 5.10), 0.2718
SkinBlood	4.39	3.65	3.87	-0.74 (-1.25 to -0.24), **0.0058**	-0.53 (-1.03 to -0.02), **0.0420**
Hannum	33.24	30.71	32.5	-2.53 (-3.53 to -1.53), **0.0001**	-0.74 (-1.74 to 0.26), 0.1664
Weidner	19.07	15.42	16.77	-3.65 (-6.68 to -0.62), **0.0190**	-2.31 (-5.34 to 0.72), 0.1509
Vidal-Bralo	62.25	60.43	62.03	-1.82 (-3.53 to -0.10), **0.0380**	-0.21 (-1.93 to 1.50), 0.9880
MiAge	663.2	661.6	668.3	-1.57 (-27.15 to 24.02), 0.9991	5.05 (-20.54 to 30.63), 0.9406
DNAmTL	6.47	6.56	6.46	0.08 (-0.02 to 0.19), 0.1201	-0.02 (-0.12 to 0.09), 0.9777

Table S5. Effect of blonanserin dose on epigenetic age of neuroblastoma cells.

	mean epigenetic age			difference (95% CI), adjusted P value	
	control	BL-low	BL-high	BL-low vs. control	BL-high vs. control
Horvath	26.61	26.89	25.31	0.27 (-2.61 to 3.16), 0.9956	-1.30 (-4.18 to 1.58), 0.5275
SkinBlood	1.66	1.58	1.31	-0.09 (-0.29 to 0.11), 0.5408	-0.35 (-0.55 to -0.15), **0.0018**
Hannum	25.12	23.34	22.92	-1.78 (-4.72 to 1.16), 0.2961	-2.19 (-5.14 to 0.75), 0.1622
Weidner	16.83	16.63	15.69	-0.20 (-2.89 to 2.50), 0.9983	-1.15 (-3.84 to 1.55), 0.5725
Vidal-Bralo	56.58	55.25	55.42	-1.33 (-2.30 to -0.37), **0.0083**	-1.16 (-2.12 to -0.19), **0.0190**
MiAge	669.8	658.3	663.9	-11.49 (-36.14 to 13.16), 0.5005	-5.85 (-30.50 to 18.80), 0.8950
DNAmTL	7.22	7.25	7.23	0.03 (-0.07 to 0.12), 0.8284	0.02 (-0.08 to 0.11), 0.9674

Table S6. Effect of perospirone dose on epigenetic age of neuroblastoma cells.

	mean epigenetic age			difference (95% CI), adjusted P value	
	control	PE-low	PE-high	PE-low vs. control	PE-high vs. control
Horvath	26.61	23.81	23.99	-2.80 (-5.69 to 0.08), 0.0573	-2.63 (-5.51 to 0.26), 0.0763
SkinBlood	1.66	1.40	1.47	-0.26 (-0.47 to -0.06), **0.0113**	-0.20 (-0.40 to 0.00), 0.0537
Hannum	25.12	23.24	23.63	-1.88 (-4.82 to 1.06), 0.2574	-1.49 (-4.43 to 1.46), 0.4356
Weidner	16.83	17.04	15.75	0.21 (-2.49 to 2.91), 0.9978	-1.08 (-3.78 to 1.62), 0.6177
Vidal-Bralo	56.58	55.48	55.03	-1.10 (-2.07 to -0.14), **0.0252**	-1.55 (-2.52 to -0.59), **0.0030**
MiAge	669.8	657.7	687.5	-12.08 (-36.73 to 2.57), 0.4606	17.71 (-6.94 to 42.36), 0.1832
DNAmTL	7.22	7.22	7.26	0.01 (-0.09 to 0.10), 0.9992	0.04 (0.05 to 0.14), 0.4823
